# Procedure to identify fortified foods in the Dutch branded food database

**DOI:** 10.3389/fnut.2024.1366083

**Published:** 2024-04-10

**Authors:** Susanne Westenbrink, Cyrelle J. Tenhagen, Ido Toxopeus, Janneke Verkaik-Kloosterman, Edith J. M. Feskens, Marga Ocké

**Affiliations:** ^1^National Institute for Public Health and the Environment, Bilthoven, Netherlands; ^2^Division of Human Nutrition and Health, Wageningen University & Research, Wageningen, Netherlands

**Keywords:** automated approach, branded foods, branded food database, decision tree, food fortification, LEDA, label data, Netherlands

## Abstract

**Introduction:**

Information on fortified foods is needed for multiple purposes, including food consumption research and dietary advice. Branded food databases are a valuable source of food label data. European labeling legislation prescribes that food fortification should be indicated in the ingredient list, and nutrient values should be declared under certain conditions. This creates the potential to identify fortified foods in branded food databases, though it is not straightforward and labor-intensive. The aim of our study was to develop an automated approach to identify fortified foods in the Dutch branded food database called LEDA.

**Methods:**

An automated procedure, based on a stepwise approach conforming with European labeling legislation, using a list of rules and search terms, was developed to identify fortified foods. Fortification with calcium, folic acid, vitamin B12, and zinc was studied as an example. The results of a random stratified sample with fortified and not-fortified foods were validated by two experts.

**Results:**

The automated approach resulted in identifying 1,817 foods fortified with one or more of the selected nutrients in the LEDA dataset (0.94%). The proportions of fortified foods per nutrient were below 0.7%. The classification of fortified/non-fortified foods matched manual validation by experts for the majority of the foods in the sample, i.e., sensitivity and specificity indicating the probability of correctly identifying fortified and non-fortified foods was high (>94.0%).

**Conclusion:**

The automated approach is capable of easily and quickly identifying fortified foods in the Dutch branded food database with high accuracy, although some improvements to the automated procedure could be made. In addition, the completeness, correctness, and consistency of the LEDA database can be improved. To fully benefit from this automated approach, it needs to be expanded to cover all micronutrients that may be added to foods.

## 1 Introduction

Healthy and safe diets providing adequate nutrient intake are essential to maintain good health. Several authors reported low intakes of micronutrients for various population groups and identified the possible contribution of fortified foods to improve intakes and related health outcomes (1–6). In a review of European evidence from 2013, it was concluded that voluntary fortification by food manufacturers can reduce the risk of sub-optimal intakes of a range of micronutrients at a population level, whereas small proportions of the population, especially children, may exceed the upper intake level for some micronutrients (6). Information on fortified foods is needed for multiple purposes, including food consumption research, personalized dietary advice, public health information, development and monitoring of food fortification strategies, and enforcement of legislation related to fortification (7–13).

In Europe, food fortification is regulated by national and European legislation to ensure safe and necessary fortification practices. Adding vitamins or minerals should at least result in significant amounts as defined by the European labeling legislation (14). On the food label, added nutrients need to be declared in the ingredient list. The total nutrient content (naturally present plus added as fortificants or other food additives) is mandatory in the nutritional panel if present in significant amounts as defined in the EU labeling legislation. Amounts are considered significant when reaching 7.5% of the dietary reference intake (DRI) for drinks per 100 mL or 15% for solid foods per 100 g and per single portion packs. Mandatory declaration of nutritional values on the label also applies in case of nutritional or health claims (14, 15). European legislation has not yet defined maximum levels for fortification. In the Dutch legislation, the maximum level is set at 100% of the reference intake (4, 16, 17), except for vitamins A and D, folic acid, iodine, selenium, copper, and zinc intakes for which fortification is prohibited to prevent excessive intake. There are, however, generic and specific exemptions for food fortification with these nutrients. For vitamin D and folic acid, a maximum of 4.5 μg /100 kcal and 100 μg /100 kcal are set as generic exemptions, respectively. In addition, for some micronutrients (e.g., zinc and copper), addition to food is allowed for restoration or substitution purposes. In the Netherlands, fortification is always on a voluntary basis, although the addition of vitamins A and D in plant-based fat products (such as margarine) and iodized salt in bread are encouraged by covenants between the food industry and the government. Legislation on food fortification in the Netherlands is summarized by de Jong et al. (4).

Most generic national food composition databases contain no or limited information on branded foods, and information on the fortification of foods is often lacking (18, 19), among others, because fortification is generally brand-specific. The growing number of branded food databases worldwide can fill this gap [e.g., (20–27)], provided that information on relevant foods, nutrient values, and fortification is present, correct, and up to date. Some authors report “manual” identification of fortified foods by experts for (subsets of) their databases, for example (11, 28–30).

The Dutch national branded food database LEDA contains food label data provided by food producers, including ingredient lists and nutritional values for energy, macronutrients, salt, and some data on micronutrients (31). The LEDA database is hosted at the Dutch Nutrition Centre. In 2022, the total number of branded foods was nearly 200,000, and it was estimated that 75% of the retail market was represented (31). The size of this branded food database and the rapid changes make identifying fortification by researchers on a food-by-food basis very labor intensive. We are not aware of any automated approach to identify fortified foods in branded food databases. Therefore, the aim of our study was to develop and validate an automated, standardized procedure to identify fortified foods in the Dutch national branded food database LEDA. Foods fortified with added calcium, zinc, vitamin B12, or folic acid were taken as an example.

## 2 Methods

### 2.1 Definition of fortified foods

To identify fortified foods, our definition is based on the European labeling legislation (EU 1169/2011). This means that we consider food to be fortified when the micronutrient, or its chemical form, is mentioned in the ingredient list and the total amount present is declared in the nutritional panel on the food label, if significant according to the European labeling legislation (14, 15). Nutrients may also be added for restoration (to make up for losses during processing) or substitution (to replicate the content of another food). European legislation does not differentiate between reasons for adding nutrients to foods, and this information cannot be retrieved from food labels. For this study, all information on added micronutrients on the food label (if complying with the labeling rules) is considered fortification. Foods and formulae for infants and young children, foods for specific medical purposes, and foods for total diet replacement often contain added micronutrients but are considered ineligible in this study because other legislation applies and EU 1169/2011 cannot be followed (4).

### 2.2 Selection of nutrients

To develop and test the automated procedure for identifying fortified foods, four nutrients were selected that can be added in multiple chemical forms. In the selection, we choose a mineral and a vitamin that can, according to the Dutch legislation, be added up to 100% of the RDA per reasonable daily consumption (calcium and vitamin B12) and a mineral and a vitamin for which addition is only allowed in lower amounts (folic acid) or specific cases [zinc is only allowed for the substitution or restauration purposes and in a specific type of menthol pastille and specific dairy products (32, 33)].

### 2.3 Procedure to identify fortified foods from the LEDA database

**Figure 1:**
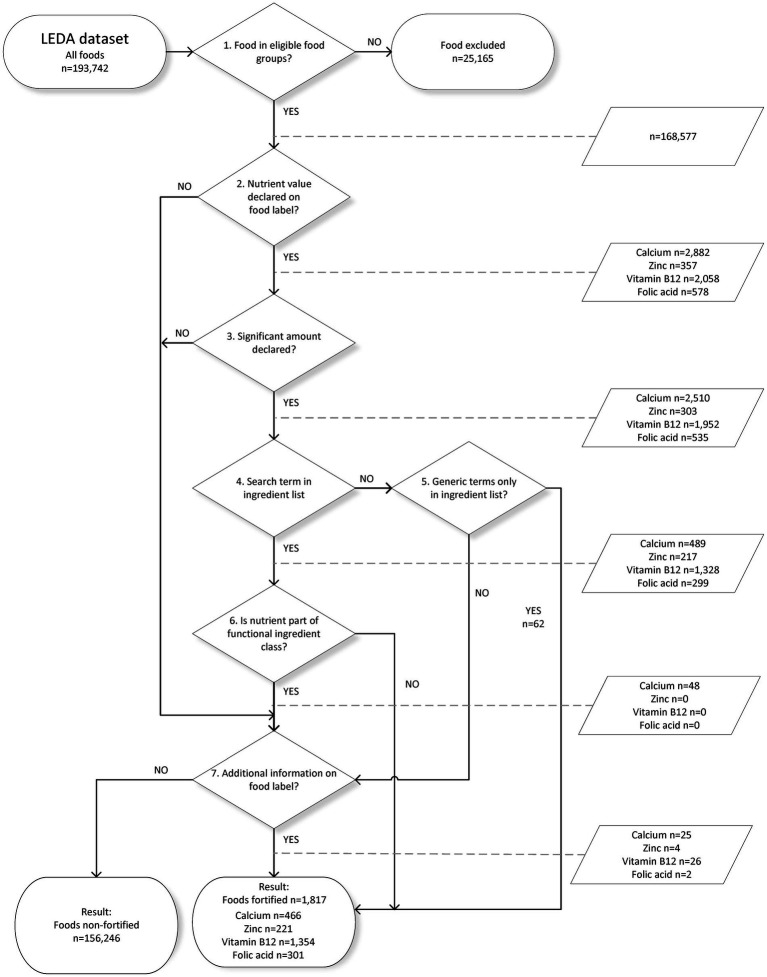
Decision tree with steps to identify fortified foods.

The European legislation on food labeling and the addition of micronutrients, as well as overarching Dutch legislation (15–17, 34, 35), was the basis for the procedure to identify if a branded food is fortified with the micronutrient(s) under study. The automated procedure is built as a decision tree (Figure 1) consisting of seven steps. In successive steps, foods are classified as ineligible, fortified, or non-fortified for each micronutrient (see Section 2.3.1). Currently, four nutrients are included, and the script can be extended to other micronutrients of interest if present in the database. For each micronutrient studied, relevant search terms need to be added. The decision tree was converted to a script in R (36).

The development of the automated procedure followed an iterative approach, particularly for the inclusion of the appropriate search terms. Vitamin formulation and mineral substances that may be added to foods were taken as the starting point (14), and ingredient lists were scrutinized for nutrient names, food additive names, and known synonyms. All search terms were in Dutch since this language is used in the LEDA database and were translated to English for this publication. Mixtures of substances are likely to be written as one word in Dutch, while in English, two separate words are used. Dutch search terms were treated as separate words by the script, resulting in all possible combinations with other compounds. The results of previous versions were verified and resulted in adaptations to improve the results. For example, searching for the wording “*fortified with”* and “*added”* not combined with specific search terms in the legal name and mandatory particulars (see EU legislation 1169/2011) in step 7 was removed as it yielded too many false positive results based on nutrients that were out of the scope.

#### 2.3.1 Decision tree

##### 2.3.1.1 Step 1: Is the food eligible?

Food groups for which legislation other than EU 1925/2006 on the addition of vitamins and minerals and other substances applies, and thus the constraints of the generic EU labeling legislation 1169/2011 cannot be followed, are considered ineligible. These included foods and formulae for infants and young children, foods for specific medical purposes, and foods for total diet replacement for weight control. In addition, food supplements and foods not classified in a food group were considered ineligible. All other foods go to step 2. Selections are based on food group classifications used in the Dutch national branded food database LEDA.

##### 2.3.1.2 Step 2: Is a nutrient value declared on the food label?

Eligible foods with nutrient values reported for the selected nutrient(s) are classified as potentially fortified and go to step 3 for additional evaluation. When nutrient values are missing, foods go to step 7.

##### 2.3.1.3 Step 3: Is the declared nutrient value significant?

The nutritional values from the potentially fortified foods, identified in step 2, were checked for significance according to the EU labeling legislation (15). It was assumed that the indicated nutrient values refer to the food in the state as sold. The legislation allows for nutritional information on the label after preparation (e.g., cooking) if clearly indicated, but the LEDA database does not give this differentiation. The cutoff values for significance differ between beverages and non-beverages and single portions (see Table 1). The definition of a beverage is not given in the legislation. We considered as beverages all foods in the group of beverages (soft drinks, juices, lemonade, water, coffee, tea, and alcohol) as well as the following subcategories of other food groups: milk, chocolate milk, condensed milk, coffee milk/cream, buttermilk, dairy drinks, and liquid breakfast based on fruit juice or dairy. The script did not differentiate between non-beverage and single-portion foods. Values equal to the cutoff value were considered significant as the legislation does not indicate that the values need to be larger than this percentage. In the LEDA database, folic acid or dietary folate equivalents (DFE) values can be reported. We decided to assume that if DFE values were available, the best option would be to consider these as an indicator of folic acid fortification. No information was available on how food producers derived DFE values. If the declared nutrient value is significant, the food is considered potentially fortified and will be further evaluated in step 4. If not significant, the foods go to step 7.

**Table 1:** Significant values for selected nutrients according to EUR-Lex (15).

Nutrient	Unit	DRI^a^	Significant amount for beverages^b^	Significant amount for non-beverages and single portion packages^c^
Folic acid	μg	200	15	30
Vitamin B12	μg	2.5	0.1875	0.3750
Calcium	mg	800	60	120
Zink	mg	10	0.75	1.50

a Daily reference intake for adults according to EU labeling legislation.

b 7.5% of the nutrient reference values are supplied by 100 g or 100 mL in the case of beverages.

c 15% of the nutrient reference values are supplied by 100 g or 100 mL in the case of products other than beverages or per portion if the package contains only a single portion.

##### 2.3.1.4 Step 4: Is the nutrient mentioned in the ingredient list?

The ingredient lists of all potentially fortified foods resulting from step 3 are searched for selected nutrients using the search terms as defined (Table 2). For foods with significant nutrient values but without any of the search terms in the ingredient list, go to step 5 for further evaluation. Foods with significant nutrient values and one of the search terms in the ingredient list were considered potentially fortified and go to step 6 for further evaluation.

**Table 2:** Search terms^a^ used (generic and for the 4 selected nutrients).

Category	Dutch search term	English search terms^b^^,^^c^^,^^d^^,^^e^^,^^f^^,^^g^
Generic	Vitamine	Vitamin
	Vitaminen	Vitamins
	Vitamines	Vitamins
	Mineralen	Minerals
	Mineraal	Mineral
	Vitamines en mineralen	Vitamins and minerals
	Vitaminen en mineralen	Vitamins and minerals
	Vitamine en mineralen	Vitamin and minerals
Folic acid	Foliumzuur	Folic acid
	B9	B9
	B11	B11
	Folaat	Folate
	Tetrahydrofolaat	Tetrahydrofolate
	Polyglytamaat	Polyglytamate
	Pfteroylmonoglutaminezuur	Pfteroyl monoglutamic acid
	Folic acid	Folic acid
	Folinezuur	
	Foliumzout	
B12	B12	B12
	Cobalamine	Cobalamin
	Cyanocobalamine	Cyanocobalamin
Zinc	Zink	Zinc
	Zinklactaat	Zinc lactate
	Zink lactaat	Zinc lactate
	Zinksulfaat	Zinc sulfate
	Zink sulfaat	Zinc sulfate
	Zinkoxide	Zinc oxide
	Zink oxide	Zinc oxide
	Zinkgluconaat	Zinc gluconate
	Zink gluconaat	Zinc gluconate
	Zinkcitraat	Zinc citrate
	Zink citraat	Zinc citrate
Calcium	Calcium	Calcium
	Calciumcarbonaat	Calcium carbonate
	Calciumfosfaat	Calcium phosphate
	Dicalciumfosfaat	Dicalcium phosphate
	Calciumlactaat	Calcium lactate
	Tricalciumcitraat	Tricalcium citrate
	Calciumcitraat	Calcium citrate
	Calciumzouten van orthofosforzuur	Calcium salt of orthophosphoric acid
	Dicalciumdicitraat	Dicalcium dicitrate
	Calciumhydroxide	Calcium hydroxide

a All search terms were in Dutch and were translated into English for the purpose of this publication.

b Capitals in the text are neglected.

c Mixtures of substances are likely to be written as one word in Dutch. The script treated the Dutch search terms as separate words, resulting in all possible combinations, e.g., calcium and zinc with other compounds.

d Combinations of calcium with another compound may be in the ingredient lists as added nutrients or as functional ingredients.

e Foods with minerals listed in combination with one of the functional ingredient classes are searched for and classified as non-fortified. For functional ingredient classes, see Table 3.

f Specific vitamins and minerals are also searched in combination with the wording mineral, vitamin and vitamin and mineral using the generic search terms for these and including delimiters as.

g Calcium is excluded when used as calcium-D-pantothenate, indicating pantothenic acid.

##### 2.3.1.5 Step 5: Is the generic term vitamin(s) and/or mineral(s) included in the ingredient list?

When specific search terms were not found in the ingredient list, the next step was to search for generic terms such as *vitamin(s)/mineral(s)/vitamin(s) and mineral(s),* not combined with any other micronutrient name in the ingredient lists (Table 2). EU labeling legislation (1169/2011) allows this generic wording when three or more micronutrients are added to the food. Foods with significant nutritional values for calcium, folic acid, vitamin B12, or zinc and one of these generic search terms in the ingredient list are classified as fortified. For the remaining foods, go to step 7 to check for additional information on the label.

##### 2.3.1.6 Step 6: Was the nutrient used as a food additive, or was the nutrient naturally present at high levels?

Micronutrients, in particular minerals, may also be used as part of food additives (e.g., stabilizers, emulsifiers, and acidity regulators) instead of fortification. The European labeling legislation requires that food additive categories be mentioned in the ingredient list, and they need to be followed by the ingredient name, which may be a chemical structure that includes one of the nutrients of interest. Of note, the legislation does not consider fortificants as a food additive category. When nutrient names (calcium, zinc, vitamin B12, folic acid, or synonyms) in the ingredient list are combined with a food additive category, e.g., antioxidant or stabilizer (Table 3), this is considered a food additive, rather than a fortificant, and these foods go to step 7 for further evaluation. Similarly, when the nutrient name is mentioned in an additional remark within the ingredient list informing the consumer that the food is a *source of [search term]* or *rich in [search term],* the food is added to the list of non-fortified foods because *source of* and *rich in* are considered to represent the natural content of the nutrient. When the specific search term is not found in combination with a food additive category or a remark about a natural high content, the food is classified as fortified food.

**Table 3:** Functional ingredient class names, according to EUR-Lex (15).

Functional ingredient class	Functional ingredient class continued
Acid	Foaming agent
Acidity regulator	Gelling agent
Anti-caking agent	Glazing agent
Anti-foaming agent	Humectant
Antioxidant	Modified starch
Bulking agent	Preservative
Color	Propellent gas
Emulsifier	Raising agent
Emulsifying salts	Sequestrant
Firming agent	Stabilizer
Flavor enhancer	Sweetener
Flour treatment agent	Thickener

##### 2.3.1.7 Step 7: Is additional information available on the food label?

Foods not identified as potentially fortified in steps 2, 3, 5, and 6 are checked for the selected search terms (Table 2) in the legal name or mandatory particulars of EU legislation 1169/2011. Foods for which selected search terms are found in combination with the wording *added* or *fortified* are identified as fortified foods. When the search terms are found in combination with the wording *source of [search term]* or *rich in [search term]*, the food is classified as non-fortified, as explained in step 6. Foods with significant nutrient values (step 3) but without any specific or generic search terms in the legal names or mandatory particulars are considered to contain natural amounts of the nutrients under study and are classified as non-fortified foods.

#### 2.3.2 Applying the automated procedure to the LEDA dataset

A data file was extracted from the LEDA database (version LEDA_20220404) in CSV format with UTF-8 encoding and contained 193,742 food items. The following variables from the database were used: food group classification (as coded by the host organization), food name, ingredient list, nutrient name, nutrient values (calcium, folic acid or dietary folate equivalents, vitamin B12, and zinc), legal food name, and mandatory particulars as provided by the food producers. Data from the food producers were considered correct. For each nutrient, the automated procedure classified each food as fortified, non-fortified, or ineligible and stored detailed information about the outcome of each step in the decision tree.

Food groups most frequently fortified with the selected nutrients are illustrated in pie charts using the food group classification shown in Table 4. To highlight the most relevant food groups for fortification, food groups with less than five fortified foods were added to the group of miscellaneous foods.

**Table 4:** Food groups in the LEDA database used to identify fortified foods.

Food group	Food group continued
Bread	Milk, milk products, and milk replacers
Bread filling	Miscellaneous
Cereals and cereal products	Nuts and seeds
Cheese and cheese substitutes	Oils and fats
Composite meals	Potatoes and other tubers
Drinks	Pulses
Eggs	Sauces
Fish, shellfish, crustacean	Snacks (sweet and savory)
Fruit	Soup
Meat replacers	Vegetables
Meat, cold cuts, and poultry	

### 2.4 Validation

Two validation steps were undertaken: a random validation for the entire procedure and a targeted validation for foods classified as fortified in step 5.

Random validation. A sample of 500 foods was taken for validation. The sample consisted of four random samples of 100 foods classified with each of the four nutrients and a random sample of the other foods (non-fortified or non-eligible foods). The results were validated by two experienced dietitians. They determined whether each food in the sample was eligible and, if so, whether it was fortified or not. The experts received specific instructions and written documentation with background information on the constraints of the EU labeling legislation. They were not aware of the outcome of the automated procedure and were not informed of the details of the decision tree. The experts worked independently from each other. The experts’ results were compared, and they were asked to reach a consensus on those foods with discrepancies in classification. The discrepancies were caused by uncertainty about whether foods were classified in the correct food groups (e.g., infant formula classified as milk product) and incorrect, unclear, inconsistent, or incomplete information in the dataset. For each of the four nutrients, a two-way contingency table was created with classes fortified, non-fortified, and ineligible foods assessed by the experts and the automated procedure. For all proportions, simultaneous 95% confidence intervals for multinomial proportions according to the methods of Sison and Glaz were calculated (37). Sensitivity and specificity were included, indicating the probability that the automated procedure correctly returned, respectively, fortified foods (true positive rate) and non-fortified foods (true negative rate). Statistical analyses were conducted in R (36).

Targeted validation. For the complete LEDA dataset, all foods were classified as fortified because a generic search term was found in the ingredient list in step 5 and was manually checked by an expert.

## 3 Results

### 3.1 Fortified foods in the LEDA dataset

For each step, the number of foods that are potentially fortified is reported in Figure 1. The final number of foods fortified with a specific nutrient can be derived by subtracting the number of “yes” for step 6 from the number of yes from step 4 and adding the number of yes from step 7. Table 5 shows the coverage of variables in the LEDA dataset and the results of the automated procedure to identify fortification with calcium, zinc, vitamin B12, or folic acid. Coverage gives the proportion of foods for which the information is available in LEDA. With 92.5%, ingredient information is considered complete for several food groups (e.g., fresh meat, fruit, and vegetables), and this information is non-mandatory. Food group classification was not yet fully added by the hosting organization (13% missing).

**Table 5:** Coverage in LEDA dataset and results of the automated procedure to identify fortification with selected nutrients.

Variable description	Data type	Coverage		Fortified	
		*n*	%	*n*	%^a^
Total number of foods		193,742	100%		
Food group classification	Text	168,577	87.0%		
Ingredient list	Text	179,243	92.5%		
Legal food name	Text	86,254	44.5%		
Mandatory particulars	Text	18,482	9.5%		
Calcium values (mg)	Number	3,741	1.9%	466	0.24%
Folic acid + DFE values (μg)	Number	963	0.5%	301	0.16%
Vitamin B12 values (μg)	Number	2,975	1.5%	1,354	0.70%
Zinc values (mg)	Number	824	0.4%	221	0.11%
Total number of foods fortified with one or more of the selected nutrients (calcium, folic acid, vitamin B12, or zinc)				1,817	0.94%

a The percentages were calculated with the total number of foods (*n* = 193,742) as the denominator.

**Figure 2:**
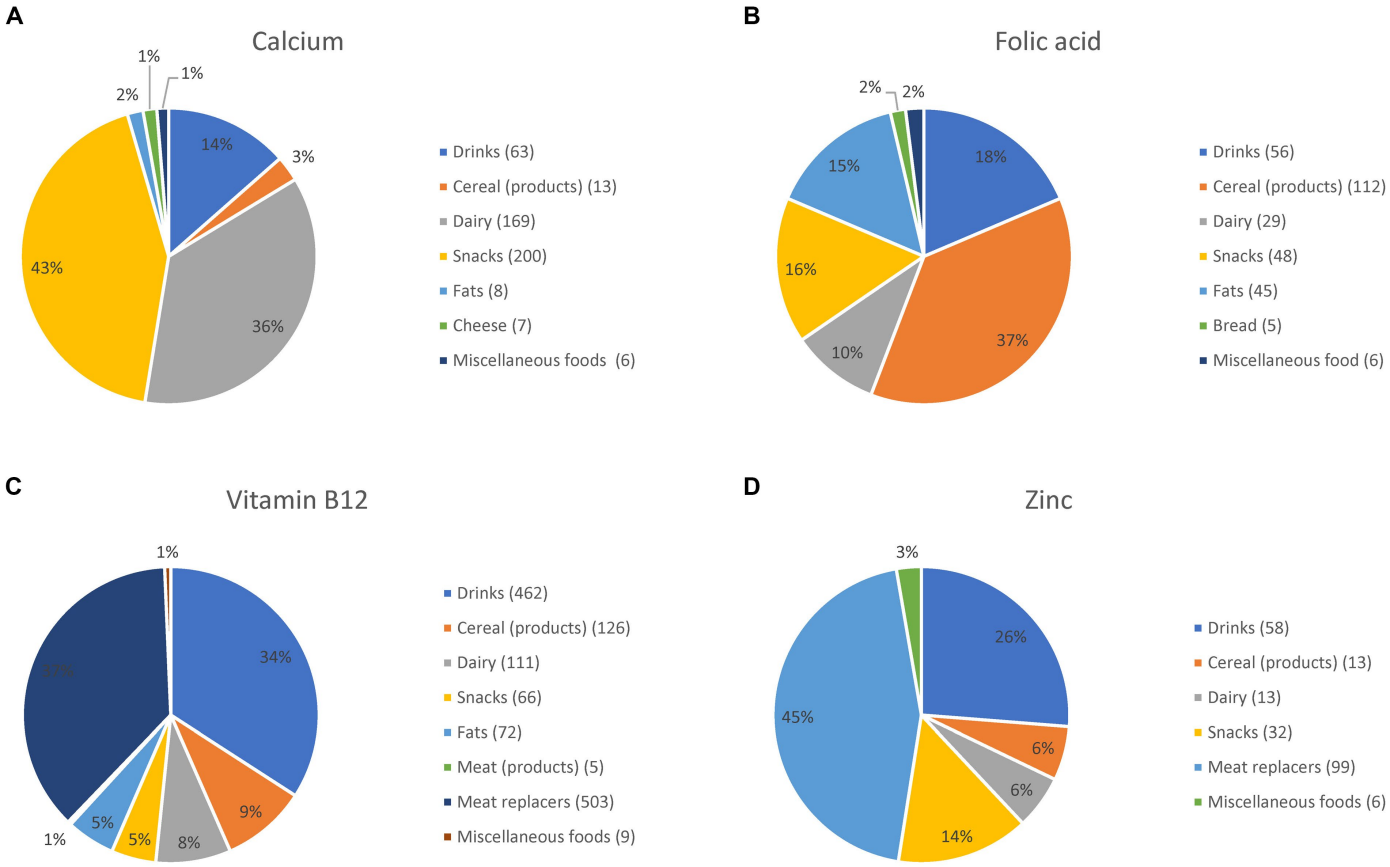
Foods fortified with calcium **(A)**, folic acid **(B)**, vitamin B12 **(C)**, and zinc **(D)** per food group; the number of fortified foods per food group is given in brackets.

The automated procedure identified 1,817 foods as fortified with one or more of the selected nutrients (0.94% of all 193,742 foods). This total percentage does not reflect the full spectrum of fortification in the LEDA database since only four nutrients were included in this study. The results per selected nutrient varied between 0.11 and 0.70% of all foods. The numbers of fortified foods per food group are shown in Figures 2A–D. For calcium fortification, dairy products and snacks (sweet snacks such as biscuits, ice cream, and sweets) were the main food groups. For folic acid fortification, cereal products were the most important food group, followed by drinks, snacks, and fats (margarine-type products). Vitamin B12 was most frequently added to meat replacers, followed by cereal products, drinks, and dairy products. Zinc was most frequently added to meat replacers (allowed in case of substitution) and to drinks (not allowed unless an exemption is given).

### 3.2 Validation of results

The comparison of the results from experts and the automated procedure for 500 randomly selected foods is shown in Table 6.

**Table 6:** Differences between experts and automated procedures for random samples of 400 fortified and 100 non-fortified foods in the LEDA dataset based on the final script.

Nutrients		Total	Procedure: fortified	Procedure: non-fortified	Procedure: non-eligible
		*n*	*n* (%) confidence interval (CI)	*n* (%) confidence interval (CI)	*n* (%) confidence interval (CI)
Calcium	Experts: fortified	106	101 (95.3%) CI: 92.5–99.2%	4 (3.8%) CI: 0.9–7.7%	1 (0.9%) CI: 0.0–4.9%
	Experts: non-fortified	379	10 (2.6%) CI: 0.8–5.0%	357 (94.2%) CI: 92.4–96.5%	12 (3.2%) CI: 1.3–5.5%
	Experts: non-eligible	15	12 (80.0%) CI: 66.7–100.0%	0 (0.0%) CI: 0.0–21.4%	3 (20.0%) CI: 6.7–41.4%
Folic acid	Experts: fortified	111	107 (96.4%) CI: 93.7–99.4%	4 (3.6%) CI: 0.9–6.7%	0 (0.0%) CI: 0.0–3.1%
	Experts: non-fortified	374	1 (0.3%) CI: 0.0–2.1%	360 (96.3%) CI: 94.7–98.1%	13 (3.5%) CI: 1.9–5.3%
	Experts: non-eligible	15	9 (60.0%) CI: 40.0–85.8%	3 (20.0%) CI: 0.0–45.8%	3 (20.0%) CI: 0.0–45.8%
Vitamin B12	Experts: fortified	253	242 (95.7%) CI: 93.7–98.1%	10 (4.0%) CI: 2.0–6.4%	1 (0.4%) CI: 0.0–2.9%
	Experts: non-fortified	232	0 (0.0%) CI: 0.0–2.9%	220 (94.8%) CI: 92.7–97.8%	12 (5.2%) CI: 3.0–8.1%
	Experts: non-eligible	15	9 (60.0%) CI: 40.0–85.8%	3 (20.0%) CI: 0.0–45.8%	3 (20.0%) CI: 0.0–45.8%
Zinc	Experts: fortified	91	91 (100.0%) CI: 100.0–100.0%	0 (0.0%) CI: 0.0–1.8%	0 (0.0%) CI: 0.0–1.8%
	Experts: non-fortified	394	0 (0.0%) CI: 0.0–1.6%	381 (96.7%) CI: 95.2–98.3%	13 (3.3%) CI: 1.8–4.9%
	Experts: non-eligible	15	12 (80.0%) CI: 66.7–100%	0 (0.0%) CI: 0.0–21.4%	3 (20.0%) CI: 6.7–41.4%

Classification of fortified and non-fortified foods by experts and the automated procedure agreed for more than 94% of the foods included. The large number of foods fortified with vitamin B12 can be explained by the large proportion of sampled foods fortified with folic acid or zinc that were also fortified with vitamin B12 (89 of 117 folic acid-fortified foods and 77 of 103 zinc-fortified foods). In the total validation sample for each nutrient, the percentages of false positive and false negative results by the automated procedure were small (0–4%), with calcium producing the most false-positive results (2.6% with 95% CI 0.8–5.0%). The false-negative results ranged from 3.6% (with 95% CI 0.9–6.7%) for folic acid, 3.8% (with 95% CI 0.9–7.7%) for calcium, to 4% (with 95% CI 2.0–6.4%) for vitamin B12. In the case of calcium, 9 out of 10 false positive results could be explained by the natural calcium content of mineral water that was mentioned (including the word calcium) in the ingredient list. It was more difficult to explain the relatively high percentage of false-negative results for vitamin B12 and folic acid. The most likely explanation is that experts made the wrong decision in cases where vitamin B12 was declared in the ingredient list and the nutritional panel, but the amount was below the level of significance. This was the case for meat substitutes, which are likely to be fortified or substituted with vitamin B12. For folic acid, in 3 of 4 cases, the decision of experts was correct, and the source of error in the automated procedure could not be identified. In the fourth case, the automated procedure and experts disagreed on whether the food was a beverage. Overall, sensitivity and specificity, indicating the probability that the automated procedure correctly classified foods as fortified and non-fortified, were high for all four nutrients. Sensitivity ranged from 96.0 to 100.0%, with all lower limits of 95% confidence intervals more than 92.5%. Specificity ranged from 94.2 to 96.6%, with all lower limits of 95% confidence intervals more than 92.4% (Table 6).

Most discrepancies between the automated procedure and experts are related to the classification of non-eligible food groups. Experts considered 15 foods as ineligible (3%) (foods and formulae for infants and young children, foods for specific medical purposes, and foods for total diet replacement), of which 12 were incorrectly classified as another eligible food group in the LEDA database. The automated procedure considered 16 foods as ineligible (3.2%), of which 13 foods were not classified in any food group in the LEDA database, whereas the experts concluded that based on the available information, the foods were eligible.

A targeted validation was done for all foods in the complete database classified as fortified based on step 5. Step 5 yielded 62 foods that were classified as fortified based on declared significant nutrient values without specific search terms but with generic search terms such as *vitamin(s)* and/or *mineral(s)* in the ingredients list. Manual checking showed that 35 of these foods were correctly classified as fortified and 27 were not, although, in some situations, information was not fully clear (e.g., incomplete ingredient lists and unexpected wording such as B(1)(2) instead of B12, that were not included as search terms).

## 4 Discussion

### 4.1 Main findings

A seven-step decision tree, aligned with European food labeling legislation, was developed to identify fortified foods in the Dutch branded food database LEDA. Steps were integrated into a script for automated application using relevant search terms. When label data correctly follows the constraints of the labeling legislation, the automated procedure successfully identifies if foods are fortified or not. Nearly 1% of the foods in the LEDA database were fortified with one to four of the micronutrients studied (calcium, folic acid, vitamin B12, and zinc). Validation showed over 94% agreement between the automated procedure and experts to identify fortified and non-fortified foods. The percentage of false-positive or false-negative results compared to the expert opinion was low (0–4%). Calcium produced the most false-positive results and vitamin B12 the most false-negative results. The remainder of the disagreements between the script and experts were for foods considered ineligible by either the script or experts (about 3%).

### 4.2 Challenges

#### 4.2.1 Data

Working with the LEDA dataset showed that identifying fortified foods is not straightforward due to the lack of specific variables to indicate fortification, complex labeling legislation, and the addition of micronutrients as food additives rather than fortification. Most challenges to developing an automated procedure were found in the LEDA data and related to the large variation in wording (including typing errors), the structure of ingredient lists, incomplete ingredient lists, wrong or missing food group classifications, data not fully in line with the European labeling legislation, and the difficulty to capture all optional search terms used in ingredient lists, legal name, and mandatory particulars. Some ingredient data were not complete or seemed to be truncated during data transfer from the food producer to the LEDA database, as complete information could be found on the food producers’ websites. As a result, some foods could not be correctly identified as fortified or non-fortified. It needs to be noted that food producers are responsible for providing complete and correct label data for the LEDA database but not for assigning the correct food classification.

Nutritional values in the LEDA dataset were supposed to be correct, but checking values was not the purpose of this study. However, errors may occur and have an impact as the values are checked for significance according to the European labeling legislation. This can be exemplified by values that were 1,000-fold too high due to decimal point or unit errors. Errors could also be related to nutritional information before and after cooking/preparing. The LEDA dataset only contained one set of nutritional values, which was assumed to represent the food as sold. However, information on nutritional composition before and after preparation is expected to become available in the LEDA database.

#### 4.2.2 Validation

For some foods, the experts’ classifications were different from the automated procedure due to their ability to combine data differently. In addition, experts had access to further information, e.g., on product websites, and this explains some of the false-positive or false-negative results.

When data aligned with labeling rules, steps 1 to 4 and 6 of the decision tree worked well. When data were less clear, steps 5 and 7 were needed. Considering all options used on labels was impossible due to the large variation in structure and wording. Currently, when generic terms such as *vitamin(s)* and/or *mineral(s)* are used in the ingredient list, legal name, or mandatory particulars, this sometimes refers to nutrients that are not in the scope of this study. This explains some of the errors found when manually checking 62 results (if yes) from step 5 and implies that the approach cannot currently be fully automated unless misclassifications are accepted. Including all nutrients that may be added is expected to limit this problem, as the generic search terms found in steps 5 and 7 will then refer to at least one of the nutrients added. As legislation may change and allow for adding other compounds containing micronutrients, updating the search terms will be needed. The option to better distinguish between the nutritional composition of raw and prepared versions of food is also expected to improve the results of the automated procedure.

Foods with significant natural levels of calcium, folic acid, vitamin B12, or zinc, and one of the generic search terms [vitamin(s)/mineral(s)] in the ingredient list due to the addition of other micronutrients would be classified as fortified if no other details were present. However, this combination did not occur in the LEDA dataset.

#### 4.2.3 Nutrients

The European labeling legislation states that nutritional values for vitamins and minerals may only be declared if significant, regardless of whether they are added or naturally present. The LEDA database also contains insignificant values provided for the database but are not shown on the food label. In combination with other information regarding added vitamins or minerals, this can complicate the decision as to whether the food should be classified as fortified, especially when judged by humans, who may tend to deviate from strict rules.

##### 4.2.3.1 Calcium

Calcium, added to fortify food, can either be mentioned in the ingredient list as *calcium* or as one of many chemical forms, e.g., *calcium carbonate, dicalcium phosphate, calcium lactate, calcium acetate,* and *calcium propionate*. Calcium is also often added for technological reasons as part of a food additive, e.g., thickener or preservative, representing various chemical forms, or it may be part of the chemical structure of vitamins, such as calcium-D-pantothenate. Food additive category names need to be declared, followed by the name of the food additive. When the food additive category is erroneously missing from the ingredient list, the food will be identified as fortified with this component if the amount is significant.

##### 4.2.3.2 Folic acid

*Folic acid* was mostly listed in the ingredient lists as such or as *vitamin B9, B11,* or *pteroylmonoglutamic acid*, and it was not found as part of food additives. According to the EU labeling legislation, folic acid may be added to foods as the synthetic form of folate or calcium-L-methylfolate. Although the total amount present in the food needs to be declared as significant, the EU legislation does not give information on how to deal with bioactivity levels of natural and synthetic forms (1.7 * natural folate). As a result, it is unclear if food producers sum folic acid and natural folate with or without conversion factors to calculate total folate of folate activity or if they only declare the amount of the added folic acid. Due to this unclearness, our procedure may interpret the significance of the values incorrectly and draw incorrect conclusions about the fortification. The US Nutrition and Supplement Facts are more clear, stating that folate and folic acid need to be declared as dietary folate equivalents (DFE) on food labels (38). Clear instructions in the European labeling legislation, as exemplified in the US, would solve this problem.

##### 4.2.3.3 Vitamin B12

Vitamin B12 can be found in the ingredient lists as *B12* or *vitamin B12* or in wordings including *cobalamin or cyanocobalamin*. Vitamin B12 was not detected as part of any food additive. Foods expected to be fortified with vitamin B12, such as meat substitutes, but with an insignificant nutritional value were confusing for experts.

##### 4.2.3.4 Zinc

Zinc is listed in the ingredient lists as *zinc* or in combination with other chemical compounds, e.g., *zinc lactate, zinc gluconate,* and *zinc sulfate*. Zinc was not found as part of food additives. In the Netherlands, adding zinc is allowed for restoration or substitution purposes only (4), and in that situation, it is not obliged to declare zinc in the ingredient list. However, it is not clear from food labels if nutrients are added for restoration, substitution, or fortification, and the automated procedure cannot make this distinction either. Although a limited number of exemptions to fortify foods with zinc are valid in the Netherlands, we found zinc added in significant amounts for many more foods and brands. Zinc added to meat substitutes may be added to substitute zinc as present in meat. Most drinks with added zinc were lemonade and vitamin water, and restoration or substation did not seem to be the reason for adding it.

### 4.3 Strengths and limitations

The main strength of our automated procedure to identify fortified foods is that it can be run quickly and as frequently as needed, with over 90% correct classification for the selected micronutrients in the validated sample. This is important because identifying fortification manually, food by food, is very time-consuming due to the size of the LEDA database and its rapid changes. In the Slovenian branded food dataset, 80% of the foods had disappeared from the market between 2011 and 2020 (27) and there is no reason to expect differently for the Netherlands. The script can easily be adapted to include additional micronutrients by adding or replacing search terms. The decision tree and the search terms can be re-used, and the script can be adapted for datasets other than the LEDA dataset. Other possible extensions of the script include estimating the amount of added micronutrients and distinguishing between the different chemical compounds of a fortificant, which can be useful for estimating bioavailability.

Another strong point was that validation was done in duplicate. Two experts independently evaluated all sampled foods and used expert opinion as the reference. Experts were flexible in combining information from multiple variables and could use additional information. On the contrary, experts were also in doubt in some cases due to the confusing, incomplete, or inconsistent information not fully complying with the rules of legislation and may have taken incorrect decisions, as exemplified by the results for vitamin B12.

A limitation of the automated procedure is that including all optional search terms is almost impossible. Although we took vitamin formulations and mineral substances that can be added to foods as a starting point and supplemented this with additional terms discovered while carefully examining ingredient lists for nutrient names, we cannot rule out the possibility that we missed some search terms. It seems more likely that we missed discovering a fortificant due to typing errors in the ingredient list. Based on the small percentages of differences between the automated procedure and expert judgment, this impact proved limited. Furthermore, fortified foods were oversampled for validation because finding fortification was expected to be more difficult than finding non-fortification. The limited sample for validation and the small number of nutrients studied only allow conclusions for the nutrients under study. Nonetheless, several options for improvement of the data and the automated procedure were identified.

### 4.4 Usability and quality of the automated procedure

Researchers may need information on fortification for food policy development, food fortification strategies, and enforcement of fortification legislation. Other use cases are personalized dietary advice and public health information. Although the incidence of fortifications in the LEDA database is low, frequent consumption of fortified food will greatly impact individual nutrient intake. The need for details may depend on the intended use; however, complete, correct, and up-to-date information on fortification, including the amounts added, is important for all users.

The validation showed that agreement between experts and the automated procedure was high (>94%), and the percentage of false-positive or false-negative results was low (0–4%). In our opinion, the automated procedure can be used to identify if branded foods are fortified. Even though there are some uncertainties and possible errors, this procedure can significantly reduce the amount of manual work needed to a manageable level. In specific situations, users may want to apply additional data-checking steps. Suggestions for improvement are mentioned under data challenges and recommendations.

Per single portion package, the European labeling legislation uses the same levels of significance as for non-beverages (15% of RDI). Calculated per 100 g or ml of food, as in LEDA, for portion sizes smaller than 100 g or ml, this results in nutritional values higher than the level of significance; for portion sizes larger than 100 g or ml, this is the other way around. Furthermore, the European labeling legislation is not fully clear if, for single-portion beverages, the RDI of 15% also applies instead of 7.5%. These limitations were not considered, as all nutritional information in the LEDA database is given per 100 g or 100 mL of food. In case of any errors in the database, the level of significance of the values may have been misinterpreted, with single-portion packages of drinks assigned higher thresholds for significance than other drinks. The LEDA dataset contained data on 113 beverages listed as single-portion packages, of which 7 were classified as fortified. The impact of possible errors was small.

Ideally, the branded food database does not contain errors in nutritional values. Automated validation, e.g., on outliers, would help to correct values during data entry. Moreover, the nutritional value in the database may deviate from the real value because food producers often add a higher amount of micronutrients to allow for losses during processing and shelf life (28, 39). Not knowing the actual amounts added or remaining and, in some cases, using incorrect values is a challenge for our approach.

In addition to the levels of significance in the EU labeling legislation, maximum fortification levels are needed to secure a safe level of intake. In Europe, maximum levels have not yet been defined, and national legislation prevails. For example, a general exemption is given in the Netherlands to fortify with folic acid to a maximum level of 100 μg/100 kcal. Checking if nutrient levels remain within the maximum level allowed is not included in the automated procedure; however, this can be monitored using the results of our approach.

If an ingredient list declares a fortified ingredient, it depends on the nutrient value (significant or not) and whether the food is considered fortified by the automated procedure. An example is the ingredient wheat flour enriched with iron, folic acid, and niacin. In the LEDA database, none of the foods containing this ingredient were classified as fortified with folic acid due to insignificant levels caused by “dilution” by other ingredients.

### 4.5 Recommendations

To improve the usability of the LEDA data to identify all fortified foods, the coverage of 75% of the food supply needs to be extended by creating liaisons with new data providers. Food group classification in the LEDA database must be completed for each food to allow the identification of fortification for all foods. Control procedures are needed to ensure that ingredient lists are uploaded without any missing information. Additional and, if possible, mandatory variables to mark fortifications at food and nutrient levels are needed both at the data provider side and in the LEDA software to allow for easier identification. Such variables could be fortified yes/no at the food level and fortified yes/no for each individual nutrient, with multiple choice options for the allowed chemical forms of food fortificants. Ideally, this would make the current approach redundant. Harmonized formats and control steps for data entry will help the food industry to improve data quality. The same applies to instructions on how to deal with substitution versus fortification on the food label. Lessons can be learned from the USDA Global Branded Food Products Database, where several so-called hard validations are in place during data entry, and if not met, further data entry is impossible (25).

To allow complete identification of fortified and non-fortified foods, all micronutrients that may be added to foods need to be added to the script. Moreover, an updated version of the script could consider significant values for single-portion foods if legal considerations are clearer.

The European labeling legislation can be further improved by (a) giving clearer information on the definition of beverages and the use of DRI for single portion packages, (b) requesting detailed information on fortification per component using dedicated variables on the food label, (c) making added micronutrients for fortification a food additive category, for which the class name needs to precede the list of individual micronutrients added, and (d) providing detailed instructions on how to define and declare the added micronutrients, in particular when conversion factors related to bioavailability are available as for folic acid and dietary folate equivalents.

### 4.6 Conclusion

A step-wise approach, including a decision tree to define if foods in the Dutch national branded food database LEDA are fortified, was developed and applied. Validation by experts showed that agreement between experts and the automated procedure was above 94% for each nutrient, and the percentages of false-positive and false-negative results were limited.

When the food label correctly followed the EU labeling legislation, the automated procedure was able to identify fortification correctly. For some foods, missing information on the label (in ingredient lists or nutritional values) led to false negative results compared to classification by experts. Inconsistent ways of presenting information on the label (wording, brackets, etc.) make it difficult to include all optional search terms, and more standardization on labels is expected to lead to better results.

To include all micronutrients that may be added to foods, the script needs to be extended. This is expected to improve results as any notification of vitamins or minerals added will then refer to one of the nutrients included in the search terms.

Considering the limitations posed by the unclear legislation and label information, this automated procedure allows quick identification of fortifications present in branded foods in the Netherlands.

## Data availability statement

The data analyzed in this study is subject to the following licenses/restrictions: data cannot be made publicly available because of contractual obligations with the data providers. Requests to access these datasets should be directed to IT, ido.toxopeus@rivm.nl.

## Author contributions

SW: Conceptualization, Methodology, Writing – original draft. CT: Conceptualization, Data curation, Writing – review & editing. IT: Data curation, Writing – review & editing. JV-K: Writing – review & editing. EF: Supervision, Writing – review & editing. MO: Conceptualization, Data curation, Supervision, Writing – review & editing.
